# *Candida glabrata* Prosthetic Joint Infection Managed with Ibrexafungerp

**DOI:** 10.3390/jof11060442

**Published:** 2025-06-11

**Authors:** Ella Nadarevic, Jack McHugh, Paschalis Vergidis

**Affiliations:** Division of Public Health, Infectious Diseases, and Occupational Medicine, Mayo Clinic, Rochester, MN 55905, USA; nadarevic.ella@mayo.edu (E.N.); mchugh.jack@mayo.edu (J.M.)

**Keywords:** *Candida*, *Candida glabrata*, prosthetic joint infection, ibrexafungerp, azole resistance, echinocandin resistance, triterpenoid

## Abstract

We report a case of a triazole and echinocandin-resistant *C. glabrata* right shoulder prosthetic joint infection in a 60-year-old woman. The patient underwent surgery and received the novel triterpenoid antifungal agent ibrexafungerp. She initially improved, but relapsed four months post-treatment. This case highlights the potential role of ibrexafungerp in multidrug-resistant *Candida* infections.

## 1. Background

*Candida glabrata* has emerged as a prominent cause of invasive candidiasis worldwide (now comprising roughly 25% of Candida bloodstream infections in the U.S.) [1]. The resistance patterns vary widely, with fluconazole resistance ranging from around 5% in certain Asia–Pacific centers to more than 20% in parts of Europe [2]. Echinocandin resistance, once under 5%, has climbed to 3–12% in recent years, owing to hotspot FKS1/FKS2 mutations [3]. Although amphotericin B remains active against *C. glabrata* [4], its intravenous route and nephrotoxicity constrain its use to salvage therapy.

Given these challenges, new oral therapies are urgently needed. Triazoles block lanosterol 14-α-demethylase, disrupting ergosterol synthesis, while intravenous (IV)-only echinocandins non-competitively inhibit β-(1,3)-D-glucan synthase at an allosteric Fks site. Ibrexafungerp (formerly SCY-078) is a novel, orally active triterpenoid that binds a distinct region of the same glucan–synthase complex, retaining activity against *Candida*, *Aspergillus*, dimorphic fungi, and even many echinocandin-resistant strains [4,5]. In 2021, the U.S. Food and Drug Administration (FDA) approved ibrexafungerp for the treatment of acute vulvovaginal candidiasis (VVC), with subsequent approval for recurrent VVC [6]. Ibrexafungerp represents the first new oral antifungal class approved by the FDA in over two decades. 

FURI was a multicenter, open-label single-arm study that evaluated the efficacy and safety of ibrexafungerp in patients with fungal diseases that were refractory to or intolerant of standard antifungal treatment. Patients were eligible for enrollment if they had proven or probable severe mucocutaneous candidiasis, invasive candidiasis, or chronic or invasive aspergillosis, with documented evidence of failure, intolerance, or toxicity related to a currently approved standard-of-care antifungal treatment. Patients who could not receive approved oral antifungal options (e.g., due to susceptibility) were also eligible, and continued IV antifungal therapy was deemed clinically undesirable or unfeasible. Here, we report a patient with a periprosthetic joint infection (PJI) secondary to azole- and echinocandin-resistant *Candida glabrata* who participated in the FURI trial and was treated with ibrexafungerp.

## 2. Case

A 60-year-old woman with compensated alcohol-related cirrhosis and bipolar disorder presented for evaluation of a right-shoulder PJI caused by *C. glabrata* resistant to both fluconazole and caspofungin.

Her orthopedic history was extensive. Six years before the index presentation, she underwent a right total knee arthroplasty that was complicated by an acute hematogenous methicillin-susceptible Staphylococcus aureus PJI, managed with a two-stage exchange. The revised joint later became infected with Streptococcus mitis and *Candida albicans*, prompting chronic suppression with doxycycline 100 mg twice daily and fluconazole 400 mg daily.

Two years before the current presentation, a periprosthetic fracture required open reduction and internal fixation of the right knee. Cultures grew *C. albicans* with dose-dependent fluconazole susceptibility, necessitating an increase in the dose of fluconazole to 600 mg daily. Subsequent recurrent knee pain led to a resection arthroplasty with hardware removal and static spacer placement. Intraoperative cultures grew fluconazole-resistant *C. albicans*. She received three months of caspofungin; suppressive antifungal therapy was not prescribed.

Fourteen months prior to the index presentation, the patient underwent a right shoulder resection arthroplasty and placement of a destination spacer for a proximal humeral fracture that had been complicated by nonunion with the subsequent development of infection. Intraoperative cultures yielded a doxycycline-resistant Staphylococcus epidermidis. She was treated with six weeks of daptomycin, followed by suppressive therapy with levofloxacin. Five months later, the patient suffered a fall and a periprosthetic fracture necessitating revision of the arthroplasty and the placement of a new destination spacer.

**Figure 1:**
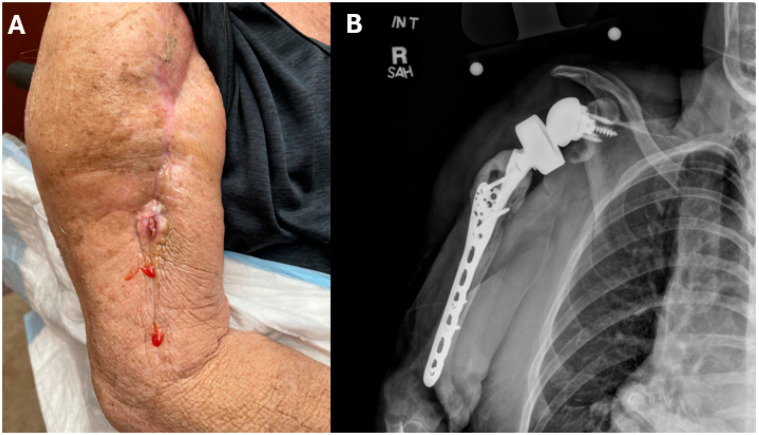
Right shoulder periprosthetic joint infection. (**A**) Image of right shoulder with purulent drainage at site of previous arthroplasty scar. (**B**) X-ray of right shoulder demonstrating significant soft tissue swelling.

Over the subsequent months, the patient reported ongoing and worsening purulent drainage from her right shoulder (Figure 1A). An X-ray of the shoulder demonstrated soft tissue swelling around the prosthesis (Figure 1B). Surgical exploration was performed. Purulence was encountered around the destination spacer. The shoulder prosthesis, proximal humeral plate, and antibiotic cement were completely removed. Cement impregnated with vancomycin and gentamicin was applied to the glenoid; an antifungal agent was not included in the cement. Five separate tissue specimens were plated on selective fungal media, with the growth of small cream colonies that were identified on MALDI-TOF as *Candida glabrata*. The patient was started on micafungin 100 mg IV q 24 h post-operatively. Itraconazole 100 mg orally twice daily was added two weeks later after the initial susceptibilities, obtained using a Sensititre^®^ Y09 microtiter plate with a colorimetric endpoint (Thermo Fisher Scientific, Waltham, MA, USA), demonstrated an intermediate susceptibility to micafungin (Table 1).

Antifungal susceptibility testing for ibrexafungerp using a broth microdilution method (CLSI M27-A) demonstrated a minimum inhibitory concentration (MIC) consistent with the epidemiological cut-off value observed in isolates without acquired resistance [7]. The patient was enrolled in the FURI trial, and, after two weeks of micafungin and a further two weeks of combination micafungin and itraconazole therapy, the treatment was changed to ibrexafungerp. A loading dose of 750 mg orally twice daily for 48 h was administered, followed by 750 mg orally once daily. A complete blood count with a differential and comprehensive metabolic panel was obtained every two weeks while on therapy. Aspartate aminotransferase increased from 28 U/L to 77 U/L after the first dose but subsequently normalized, and no other laboratory abnormalities or adverse events were noted while on therapy. The patient received ibrexafungerp for 15 weeks. The surgical wound healed completely, and she experienced the resolution of her right shoulder pain.

Four months after stopping ibrexafungerp, the patient experienced recurrent drainage at the shoulder arthroplasty site. Surgical exploration was performed. Necrotic debris was noted around the destination spacer. A Steinmann pin was inserted as a temporary spacer to help maintain alignment. High-dose antibiotic-laden cement including amphotericin B, vancomycin, and gentamicin was applied throughout the spacer, and a wound VAC was placed. Four separate tissue specimen fungal cultures grew triazole- and echinocandin-resistant *C. glabrata*, and two bacterial tissue culture specimens grew Enterococcus faecalis. The MIC of ibrexafungerp was unchanged at 1 µg/mL (Table 1). Another course of ibrexafungerp was administered through the expanded access program, and the patient received piperacillin–tazobactam for an E. faecalis co-infection.

One month later, further drainage from the shoulder was noted, and Enterococcus faecalis was again isolated. Further orthopedic intervention was deemed unsafe. A family discussion led to a transition to home hospice and cessation of antimicrobial therapy, and the patient died two months thereafter.

## 3. Discussion

As a first-in-class triterpenoid antifungal targeting β-(1,3)-D-glucan synthetase, ibrexafungerp has demonstrated potent in vitro activity against a broad range of *Candida* spp. In the FURI trial, among 233 patients with *Candida* infections, 41% achieved a complete response, while an additional 39% showed a partial response or stable disease [8]. Notably, ibrexafungerp was well tolerated, with no significant hepatotoxicity or nephrotoxicity reported in the trial or prior studies [5]. This case represents the first reported use of ibrexafungerp for a Candida PJI and highlights both its promise and limitations in managing deep-seated fungal infections. Several key considerations emerge from this case.

First, the presence of both fluconazole and echinocandin resistance at the time of initial infection was a significant complicating factor. Over several years, the patient received chronic suppressive fluconazole therapy, initially at standard dosing and later at an increased dose in response to *C. albicans* with dose-dependent susceptibility. This prolonged azole exposure likely exerted selective pressure favoring *C. glabrata* strains harboring resistance mechanisms, such as the upregulation of ATP-binding cassette transporters (CDR1, CDR2) and ERG11 mutations, conferring fluconazole resistance [9]. Similarly, repeated courses of echinocandins, including caspofungin and micafungin, may have contributed to FKS-mediated resistance [10]. This well-described mechanism in *C. glabrata* involves mutations in FKS1 or FKS2, reducing the β-(1,3)-D-glucan synthase susceptibility to echinocandins. Ibrexafungerp remained active, however, likely due to its distinct binding site on β-(1,3)-D-glucan synthase, which differs from that of echinocandins. Prior studies support this observation; one investigation of 89 *C. glabrata* bloodstream isolates demonstrated that ibrexafungerp retained activity against all echinocandin-resistant strains, despite the presence of FKS mutations [11].

The patient’s initial favorable response highlights the role of ibrexafungerp as a viable therapy. However, multiple factors likely contributed to the eventual relapse. *Candida* PJI carries an inherently high risk of treatment failure, with the relapse rates exceeding 40% in large series [11]. Biofilm formation on prosthetic materials represents a major barrier to eradication, and, in this case, the patient’s cirrhosis and history of recurrent *C. albicans* PJIs suggested an impaired immune response. Cirrhosis has been associated with a two to five-fold increase in invasive candidiasis [12]. Although the surgical approach was extensive, prior interventions on the shoulder and chronic inflammation likely provided a favorable environment for persistent infection.

Polymicrobial involvement further complicated management. At the time of relapse, *Enterococcus faecalis* was isolated, raising the possibility that the bacterial co-infection facilitated fungal persistence. While ibrexafungerp maintained activity against the patient’s *Candida* isolates, deep-seated infections and incomplete surgical clearance can limit antifungal efficacy. Even novel antifungals such as ibrexafungerp may not fully overcome these host- and pathogen-related barriers. In retrospect, a prolonged course of ibrexafungerp (e.g., 6–12 months) might have provided more sustained control, in line with the Infectious Diseases Society of America’s guidelines recommending prosthesis removal followed by at least three months of antifungal therapy for *Candida* PJIs, with longer durations in immunocompromised individuals [13]. 

Nonetheless, ibrexafungerp occupies an important niche for patients with limited intravenous access, drug intolerance, or multidrug-resistant *Candida* isolates. Its oral formulation may improve adherence, and data from the FURI trial support its role in refractory infections. However, this case highlights the reality that relapse remains a concern in *Candida* PJIs, even with novel agents. Ultimately, optimal outcomes depend on careful patient selection, aggressive surgical management, and close post-treatment surveillance.

## 4. Conclusions

Ibrexafungerp expands the antifungal armamentarium for difficult-to-treat *Candida* infections. However, clinical experience remains limited, and further data are needed to define its optimal role. Until then, ibrexafungerp should be viewed as a helpful but not failproof resource for patients in whom conventional options have been exhausted.

## Figures and Tables

**Table 1:** Susceptibility profile of *Candida glabrata* at index presentation and at time of recurrence.

Antifungal	Index Surgery MIC (µg/mL)	Recurrent Infection MIC (µg/mL)	CLSI Breakpoint (µg/mL) Susceptible	CLSI ECV (µg/mL) Wild-Type
Amphotericin B	1	0.5	-	≤2
Anidulafungin	0.25	0.5	≤0.12	-
Caspofungin	1	2	≤0.12	-
Micafungin	0.12	0.25	≤0.06	-
Fluconazole	128	256	≤32 ^1^	-
Itraconazole	1	>16	-	≤4
Posaconazole	2	>8	-	≤1
Voriconazole	1	4	-	0.25
Ibrexafungerp	1	1	-	-

MIC: minimum Inhibitory concentration; CLSI: Clinical Laboratory Standards Institute; ECV: epidemiological cut-off value; ^1^ susceptible dose-dependent.

## Data Availability

Data are not publicly available.

## Abbreviations

The following abbreviations are used in this manuscript:

IV	Intravenous
PJI	Prosthetic Joint Infection
MALDI-TOF	Matrix-Assisted Laser Desorption/Ionization Time-of-Flight (Mass Spectrometry).
MIC	Minimum Inhibitory Concentration
